# The collective voice of early phase COVID-19 vaccine trial participants: Insights for improving confidence in novel vaccines

**DOI:** 10.1080/21645515.2023.2203023

**Published:** 2023-05-03

**Authors:** Tonia M. Thomas, Susanne H. Hodgson, Katherine Emary, Maia Patrick-Smith, Rebecca te Water Naude, Arabella S. V. Stuart, John Henry, Marcus English, Maria Moore, Naomi Douglas, Andrew J. Pollard, Samantha Vanderslott

**Affiliations:** a Oxford Vaccine Group, University of Oxford - Old Road Campus, Oxford, UK; b Jenner Institute, University of Oxford - Old Road Campus, Oxford, UK; c Medical School, University of Oxford - Old Road Campus, Oxford, UK; d Department of Paediatrics, University of Oxford, Oxford, UK

**Keywords:** Vaccine trial, COVID-19, participants, confidence

## Abstract

In early 2020, adult volunteers were invited to participate in a first-in-human trial of the COVID-19 vaccine, ChAdOx1 nCoV-19, in the United Kingdom (UK) at the height of the global pandemic when there was uncertainty regarding vaccine efficacy and side-effects. We conducted a retrospective survey of these uniquely situated individuals to gain insight into their views about the risks, motivations, and expectations of the trial and potential vaccine deployment. Our data from 349 respondents show that these volunteers were educated to a high-level with a clear understanding of the seriousness of the COVID-19 pandemic, as well as an appreciation of the role of science and research in developing a vaccine to address this global problem. Individuals were primarily motivated with altruistic intent and expressed a desire to contribute to the scientific effort. Respondents appreciated that their participation was associated with risk but appeared comfortable that this risk was low. Through our analysis, we highlight these individuals as a group with strong levels of trust in science and a sense of societal responsibility, and therefore are a potential valuable resource to improve confidence in novel vaccines. Vaccine trial participants could offer a credible collective voice to support positive messaging around vaccination.

## Introduction

The University of Oxford’s COV001 clinical study in 2020, was the first-in-human Phase I/II trial of ChAdOx1 nCoV-19 (Oxford-AstraZeneca vaccine, Vaxevria).^1^ The trial was performed under unusual circumstances, when the United Kingdom (UK) was in lockdown during a global pandemic, with high media and public interest due to the necessity of accelerated vaccine development to control COVID-19. Therefore, the individuals who took part in the study formed a unique cohort, set apart from those who typically take part in vaccine trials in non-pandemic times.^2^ Previous studies on those who were willing to take part in vaccine trials (both in actuality and hypothetically) in non-pandemic cases have explored motivations and barriers for enrollment.^3^ Such studies have identified altruism and the desire for social benefits as key motivators, as well as personal benefits of personal protection against disease, which can be related also to psychological and financial wellbeing. The barriers to taking part in trials have related principally to safety concerns and a fear or mistrust or the trial and those organizing and funding the research.^4^ Other concerns have been connected to concerns or misunderstandings about study design (especially when these were complex and used unclear technical terms) and worries about discrimination and social risk, as well pragmatic obstacles such as inconvenience and disruption of daily activities.

While such research is informative, there has been a concentration on analyzing participation on vaccine trials for particular diseases such as HIV/AIDs, highlighting the importance of conducting similar studies about participants across vaccine studies and contexts. We are interested in what drove COVID-19 vaccine trial participants to come forward and participate when others did not, their reasons for participation, their experience of taking part during the pandemic, and their views about vaccination more broadly. The objective of this mixed-methods study completed alongside the COV001 trial is to explore what can be learned from this unusual phase I/II study cohort and the context of their participation, offering wider insights into vaccine trial participation, including for future pandemics.

## Methods

### COV001

COV001 (NCT04324606), is a UK multicentre, first-in-human, single-blinded, randomized Phase I/II study of COVID-19 vaccine: ChAdOx1 nCoV-19. This study included healthy volunteers aged 18–55 years. Recruitment for the study started in March 2020 and the first volunteers were enrolled in April 2020.^1^ Recruitment and eligibility criteria for the study have been published previously. The participant information sheet for the study is available in supplementary materials.

### COVQUAL

Participants in COV001 who had attended a screening visit for participation in COV001 at the Oxford site and had consented to be contacted about future vaccine-related research (*n* = 771) were invited to participate in a mixed-methods study emphasizing qualitative approaches (COVQUAL) to understand individuals’ motivations for volunteering for COV001 and their perception of the risk of participation. Invited individuals included participants who had been enrolled in the trial (i.e. those vaccinated) and those who were excluded following screening. The COVQUAL study was independent of COV001. The study design, survey questions and protocol were approved by the University of Oxford Medical Sciences Division Ethics Committee (Ref: R70147/RE001) prior to the start of the study. All participants in COVQUAL provided written informed consent.

### Survey design

The online survey contained multiple discrete sections and is detailed in the Supplementary Information. Participants were asked to provide the following categorical demographics: sex, age, nationality, ethnicity, education, employment, occupation, income, living arrangements, marital status, children, and religion. Participants were also asked if they had been financially disadvantaged by the pandemic and whether they had previously participated in a clinical trial. Individuals were not required to provide identifiable information at any point.

The survey then used a five-point Likert scale to assess participants’ agreement to statements addressing their motivations to volunteer; their perception of risk; and their views on the potential outcomes of the trial. Participants could add free-text comments to their survey responses, which is in keeping with previous studies.^5^ The content and clarity of the survey was assessed by the Oxford Vaccine Group’s Patient and Public Involvement group and edited following their feedback prior to use.

### Survey distribution

The survey was delivered using JISC Online Surveys. The survey was open for 4 weeks and a reminder e-mail was issued 2 weeks after launch to prompt any participants who had not yet completed the survey.

### Data analysis

Responses to survey statements were coded numerically for data processing: Strongly disagree = −2; disagree = −1; neither agree nor disagree = 0; agree = 1; strongly agree = 2. Where appropriate, responses were collated and used to calculate median scores.

Statements regarding motivations for taking part in the study were retrospectively grouped into personal or societal motivations following an unbiased correlation analysis, using Kendall’s Tau correlation coefficient. Responses were then collated to generate a median score for societal motivation and personal motivation.

Correlations were assessed using Kendall’s Tau correlation coefficient. Significance testing of an association between groups was completed using the non-parametric Kruskal–Wallis chi-squared test. Two tailed p-values *<0.05* were considered statistically significant.

Data analysis was conducted in Microsoft Excel (version 16.46), and RStudio (version 1.3.959). (Team (2018). RStudio: Integrated Development for R. RStudio, Inc., Boston, MA).

## Results

A total of 349 participants from the COV001 vaccine trial completed a survey to assess their motivations for taking part in the trial and their attitudes about the risks and benefits of participating. This was the full completion number, with 349 surveys filled out completely with no question left unanswered (only surveys with all questions completed were able to be submitted), and we did not set a cutoff for the percentage of survey completion for the surveys to be included in the analysis. The findings offer a relevant and uniquely situated public perspective on individuals’ perception of the risks and benefits of participation in clinical trials and vaccination more widely. Findings are grouped into three key areas: (1) motivations for participation, (2) perception of risk, and (3) trust in science and research.

In September 2020, participants of the COV001 trial were invited to complete a retrospective survey, and 43% (349/812) responded. All survey participants included in the analysis had received a vaccine in the trial (blinded to either the novel COVID-19 vaccine or the control vaccine), and no efficacy results had been announced at the point of data collection. Respondents were evenly split by sex (female 55%, 191/349), and the median age group was 45–55 years (33%, 114/349) (See Supplementary Data for more detailed demographic data). More than half of respondents (56%, 194/349) were educated to postgraduate level and were employed full time 56% (197/349), with education, law, and government services being the most commonly reported occupational groups. Forty percent (138/349) were living with a partner, 50% (173/349) were single, and 62% (216/349) did not have children.

### Motivations for participation

**Figure 1.**
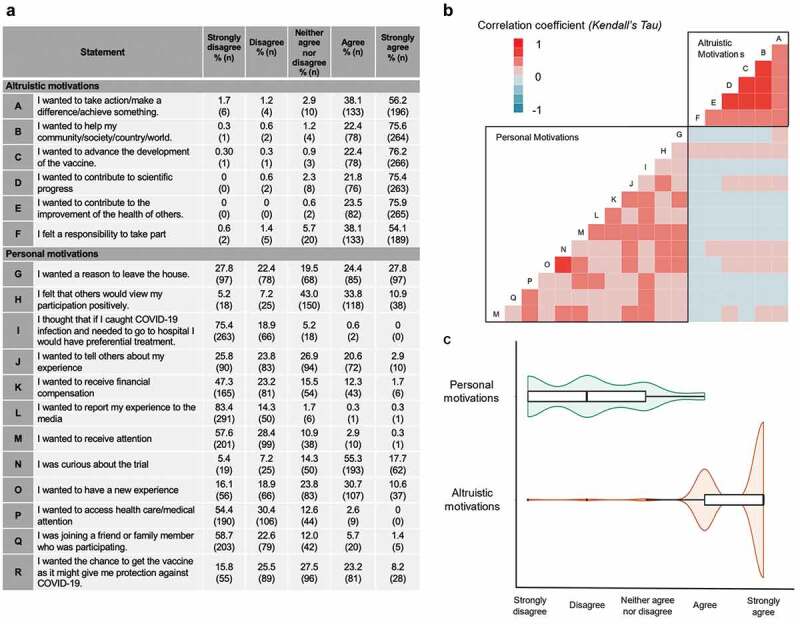
Respondents’ motivations for participating in COV001. (a) Respondents’ agreement with statements regarding altruistic and personal motivations. (b) Correlation between participants responses to individual altruistic and personal motivation statements (Kendall’s Tau). (c) Spread of responses to altruistic and personal motivation statements.

Respondents’ motivations for participating in the COV001 vaccine trial were assessed by asking their agreement to statements that were classified as ‘altruistic’ motivations that benefited the collective or ‘personal’ motivations that benefitted the individual personally (Figure 1). Most participants agreed that altruistic motivations, such as wanting to help their community (“agree or strongly agree” = 98%, 342/349), wanting to advance the development of a COVID-19 vaccine (“agree or strongly agree” = 98.6%, 344/349), and wanting to contribute to the improvement of the health of others (“agree or strongly agree” = 99.4%, 347/349) were strong motivating factors.

Responses to survey statements were coded numerically for analysis: Strongly disagree = −2; disagree = −1; neither agree nor disagree = 0; agree = 1; strongly agree = 2. For violin plot, thick black line inside boxes indicates median, box edges indicate lower and upper quartiles. Violin shape indicates proportion of data around each response.

Most participants disagreed that personal benefit was a motivation for taking part in the study, and only 31% (109/341) agreed that the opportunity to be vaccinated and acquire personal protection against COVID-19 was an important motivator to volunteer. Half of respondents (52%, 182/341) agreed that having a legitimate reason to leave their home in lockdown to attend study clinic visits was an important motivator for participation. Of interest, 73% (255/349) agreed that curiosity regarding the clinical trial was also an important motivator. The majority of respondents (71%, 246/341) disagreed that the financial reimbursement offered for participation in the study (£235–625) was a personal motivator.

A correlation analysis was used to identify patterns between individual responses to statements about motivation for taking part. Strong correlation was seen between individuals’ survey responses within the groups of ‘altruistic’ and ‘personal’ motivations, with limited correlation in responses between these two groups (Figure 1b). When considered collectively, statements referring to ‘altruistic’ motivations showed a median response of “strongly agree” whilst those referring to ‘personal’ motivations showed a notably broader distribution, with a median response of “disagree” (Figure 1c). Comments volunteered by respondents suggested that altruistic motivations did not simply refer to the participants’ community or nation but to the wider global community, with participants referring to ‘*the children’*, ‘*the planet’*, ‘*anyone*’ and ‘*vulnerable people everywhere’* (Box 1).

**Table:** **Box 1.** Participants’ comments regarding their motivations for participating in the COV001 vaccine trial.

*“I honestly just wanted to do my bit. I work with young children and I see daily how much this pandemic has affected them and I truly believe that without a vaccine, that won’t improve.”*
*“I just wanted to do my very small bit to help the planet.”*
*“I lost a family member to COVID shortly before my vaccination and would not wish the experience of losing someone during this pandemic on anyone.”*
*“My reason for joining the trial was simple: I wanted to contribute in my own way to find a vaccine to defeat a global pandemic. We, the volunteers, are here because we recognize the importance of a vaccine breakthrough and for it to be made freely available for vulnerable people everywhere.”*

**Figure 2.**
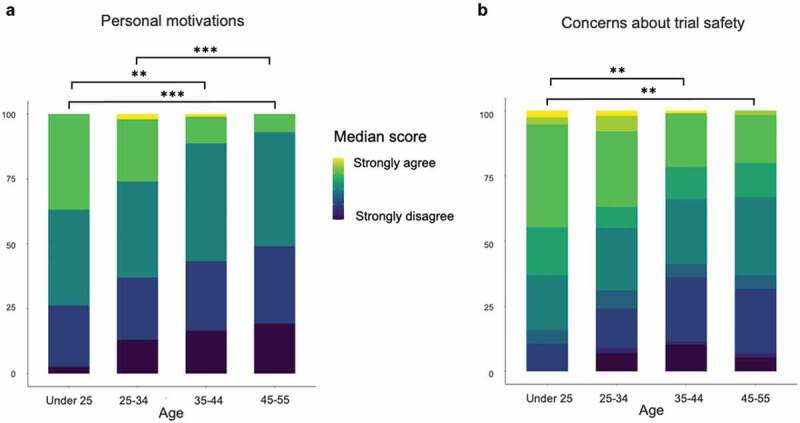
How participants’ age affected their motivations and concerns about participating in COV001. (a) Younger people (under 25 and 25–34) were more likely to have personal motivations than older people. (b) Younger people (under 25) also reflected higher levels of concern about trial safety.

Individuals under 25 years old and aged 25–34 were more likely to agree with statements suggesting personal benefit were a motivation for participation than older age groups (Figure 2a). Those in the under 25 age group were also more likely to agree with statements suggesting they had some concerns about the safety of the trial (Figure 2b).

Data shown as percentages. Significance: Kruskal–Wallis chi-squared test, ** = *p* < .01, *** = *p* < .001.

### Perception of risk

**Figure 3.**
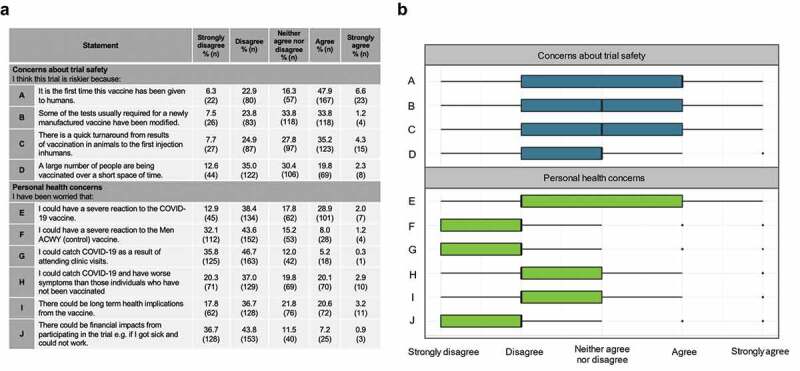
Participants’ perceptions of risk relating to the trial: (a) Proportions of participants in agreement with statements regarding risk of participating in COV001. (b) Participant responses to statements regarding risk of participating in COV001. Responses to survey statements were coded numerically for analysis: strongly disagree = −2; disagree = −1; neither agree nor disagree = 0; agree = 1; strongly agree = 2. For box plot, thick black line indicates median, box edges indicate lower and upper quartiles.

Although the vaccine being tested was similar to an MERS vaccine previously tested in humans, the COV001 study could be perceived as higher risk than previous vaccine studies due to the novel nature of the SARS-CoV-2 virus. As well as being a first-in-human trial, the number of study participants increased more rapidly than other vaccine studies which may have resulted in more caution from participants. All participants were aware of the risks associated with the trial via participant study documents and had provided informed consent before taking part. Upon questioning about their perception of trial-associated risks, participants varied in their responses but did appear to feel strongly that the study was higher risk by virtue of its design (Figure 3).

Participants were questioned about their concerns regarding the risks of participation. A notable proportion of participants disagreed that they were concerned about the potential of (i) vaccine-induced enhancement of COVID-19 disease (disagree or strongly disagree = 57%, 200/349 participants), (ii) severe reaction to the COVID-19 vaccine (disagree or strongly disagree = 51%, 179/349 participants), or (iii) the possibility of long-term health complications following vaccination (55%, 190/349 participants), despite all these potential complications being highlighted during study enrollment as potentially severe, although unlikely, risks following vaccination.

A number of participants volunteered their own thoughts on risk with one individual commenting, “*I felt compelled to participate as the risk of doing nothing to help seemed higher*,” suggesting that this participant appraised the risks of inaction in context of the early COVID-19 pandemic to be higher than risks of participating in COV001. Another participant commented, “*The trial is conducted most transparently and with respect and care for the participants. I felt like part of the team! Risk belongs to normal life*.” This response suggests the participant appreciated clear discussion of risk but was also able to contextualize this risk and appreciate that risk was not unique to COV001 and rather an intrinsic part of everyday life.

One participant stated, “*I have trust in the researchers and the ethical review process, so it has always felt like a small manageable risk*,” suggesting the reputation of the investigators and the participant’s knowledge of the review processes surrounding clinical trials helped mitigate the perceived risk of participation.

### Trust in science

**Figure 4.**
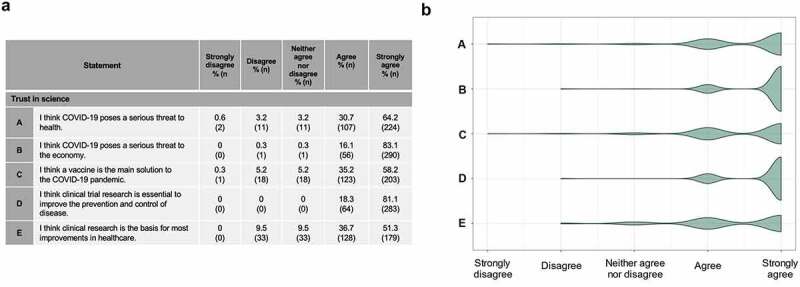
Respondents’ trust in science (a) Respondents’ agreement with statements regarding COVID-19 and science. (b) Spread of responses to statements assessing trust in science.

Participants were asked about the role they felt research and a vaccine would play in the COVID-19 pandemic (Figure 4). Respondents strongly agreed, even at this relatively early stage of the pandemic, that COVID-19 was a serious global threat and that a vaccine would be the main solution to the pandemic *(agree or strongly agree = 93% (326/349)*. Respondents also agreed that clinical trial research is essential to improve the prevention and control or disease *(agree or strongly agree = 99% (347/329)*.

Responses to survey statements were coded numerically for analysis: Strongly disagree = −2; disagree = −1; neither agree nor disagree = 0; agree = 1; strongly agree = 2. Violin shape indicates proportion of data around each response.

There was a strong correlation between altruistic motivations and trust in science and research (*R = 0.29, p < .001*), with 96% of participants either agreeing or strongly agreeing to both sets of questions. Nearly all respondents reported the desire to contribute to scientific advancement as a motivation for volunteering (*agree or strongly agree* = 97%, 339/349) (Figure 1). Faith in science and research further translated into outcome expectations for the COV001 study, as 65% (227/341) of participants agreed or strongly agreed that the COVID-19 vaccine “*will work*,” with unsolicited comments such as “*research is the only way out*.” There were no other associations between demographic details and trust in science.

## Discussion

This research provides an insight into the motivations, views, and experiences of individuals volunteering to participate in a ‘first-in-human’ UK early-phase COVID-19 vaccine trial, early in the COVID-19 pandemic. At that time, much was unknown regarding SARS-CoV-2, with no available treatments. The ChAdOx1 nCoV-19 vaccine was the first vaccine candidate to enter clinical trials in Europe, with the Moderna vaccine beginning clinical testing approximately one month before. Whilst clinical data for previous experimental viral-vectored vaccines failed to show any safety concerns, ChAdOx1 nCoV-19 was untested in humans with the unlikely but theoretical potential to enhance COVID-19 disease. Given that the SARS-CoV-2 virus was only detected 4 months prior to participants applying to take part in COV001, individuals volunteering for this trial did so in unusual circumstances.

Other research has focused on COVID-19 trial participants in the United States (US) and the role of individual rights vs collective responsibility, as well as their experiences of receiving a vaccine using semi-structured interviews.^1^ Our earlier publications using this survey and interview dataset specifically concentrated on views about animal testing and vaccine nationalism.^6,7^ More limited research has taken place on populations in low- and middle-income countries,^3^ but COVID-19 vaccine trial participation research is lacking. Our data show that volunteers in UK vaccine trials were educated to a high-level with a clear understanding of the seriousness of the COVID-19 pandemic and appreciation of the role of science and research in developing a vaccine to address this global problem, regardless of their age, occupation, or educational background. Individuals were primarily motivated with altruistic intent and expressed a desire to contribute to the scientific effort. Respondents appreciated their participation was associated with risk but appeared reassured this was low.

Younger people under 25 years old were more likely to be motivated by the potential personal benefits of participation in COV001, for example financial reimbursement or a desire for a legitimate reason to leave home in lockdown. This group also had more concerns about trial safety, although not enough to deter their participation. This suggests that younger individuals may need to be targeted more specifically with regard to vaccine research and safety messaging, particularly given this cohort is at low risk of severe disease following COVID-19 infection. Our profiling of individuals who volunteered to take part in an early-phase COVID-19 vaccine trial provides information toward targeted recruitment of volunteers for similar trials in future outbreak scenarios but more significantly, we show that these individuals may be a valuable resource. As a group with strong levels of trust in science and a sense of societal responsibility, vaccine trial participants can offer a credible collective voice to support positive messaging around vaccination.

Throughout the pandemic, media and public scrutiny of vaccine trials has been prevalent, particularly focused upon vaccine safety, which has been shown to influence desire to vaccinate.^8^ False news is known to spread faster and to more people than the truth, particularly false stories that instill fear, disgust or surprise.^9^ Such narratives on social media can spread virally and are disseminated more widely than traditional media. For COVID-19 vaccines, despite reassuring data, safety concerns have been cited as reasons for ongoing hesitancy regarding vaccination,^10^ particularly in lower-income countries, where fewer opportunities arise for vaccine developers to engage in counter-conversations regarding vaccine safety.^11^

While the voice of vaccine trial participants has been in high demand throughout the pandemic, with many appearing in media interviews,^6^ the focus of such interviews has typically focused on their individual experiences, rather than their views on risk, vaccine side-effects, or countering misinformation. Relatable narratives and experiences may be more effective than scientific facts in influencing decision-making,^12^ making the collective voice of participants a valuable asset for public health strategies. Indeed, celebrity endorsement of COVID-19 vaccines from relatable figures, as was seen in the UK and US, was implemented to garner public support for COVID-19 vaccine roll-out programs.^13^

Relaying vaccine trial participants’ support for research, altruistic motivations, and low perceived risk of taking part could offer a vital tool in overcoming misinformation and instilling confidence in vaccine development. For example, respondents in our study accepted that participation in the COV001 vaccine trial was associated with some risk, however, were willing to accept this and volunteer nonetheless: “*Risk is part of life*.” It is likely that such a message from a trial participant may carry more weight than if delivered by a politician or even a scientist.

We acknowledge that there are some limitations in the design of this study, which included not using a validated checklist for the survey. While we have ensured that many of the principles of survey research have been applied to reduce bias^14^ and enhance rigor, there have been several limitations in conducted a rapid research project during the COVID-19 pandemic. Due to timeliness and the novelty of the research project, we were not able to conduct pre-survey piloting and testing on a systematic scale. While our survey’s face value was established by experts, it was only pilot tested within our team and not with a subset of participants as we were using a purposeful sample, and therefore wanted to use all the results from this group. As a result, the survey may not provide the same level of objective robust data that would be expected through validation.

There are some questions for example, that may have had a wide interpretation. Asking participants if they were “financially disadvantaged by pandemic?” could have been understood in different ways, such as from losing work (e.g. being furloughed), or having to work less (e.g. reducing work to part-time hours), as well as additional responsibilities and costs (e.g. childcare). The relatively high proportion of respondents who answered “yes” or “prefer not to say” for this question may also have been due to the characteristics of those able to participate in a vaccine trial, because of the time needed to attend appointments whilst taking part or being incentivized by the financial reimbursement for participation.

We also note that our respondents are not representative of the wider population and there are some risks in giving a greater voice to vaccine trial participants. Trial participants have often taken part in other trials before and so may have a greater comfort and confidence in the trial process, which differs from the general public. For example, one method for recruiting trial participants was via a participant database and newsletter. Also, the trial was advertised via the Oxford Vaccine Group website and social media channels. Therefore, a high number of participants who had previously taken part in a clinical trial were to be expected, answering the question: “have you participated in a clinical trial before?.”

Furthermore, while a trial is ongoing, participants should not jeopardize the integrity of the trial and may not be best equipped to cope with a communication role and the attention this may attract. However, a greater visibility of their views and involvement would help to improve the openness and transparency of vaccine trials, as well as setting an example to reinforce public health messages. If carefully managed by researchers alongside the clinical study team, obtaining and publicizing collective views would be possible without risking trial integrity.

Public engagement campaigns and qualitative studies in parallel with vaccine trials could provide relatable voices from participants who have considered the risks and benefits of vaccination carefully and have an “*insider perspective*” of research. Our data must be caveated by the unusual circumstances in which COV001 was undertaken. However, vaccine participants may offer a well-informed and relatable collective voice that could provide a powerful addition to messaging campaigns, particularly regarding vaccine safety.

## Supplementary Material

Supplemental Material

## References

[1] Folegatti PM, Ewer KJ, Aley PK, Angus B, Becker S, Belij-Rammerstorfer S, Bellamy D, Bibi S, Bittaye M, Clutterbuck EA, et al. Safety and immunogenicity of the ChAdOx1 nCoV-19 vaccine against SARS-CoV-2: a preliminary report of a phase 1/2, single-blind, randomised controlled trial. Lancet. 2020;15396(10249):467–7. doi:10.1016/S0140-6736(20)31604-4. PMC7445431 32702298

[2] Wentzell E, Racila AM. The social experience of participation in a COVID-19 vaccine trial: subjects’ motivations, others’ concerns, and insights for vaccine promotion. Vaccine. 2021;39(17):2445–51. doi:10.1016/j.vaccine.2021.03.036. 33745730 PMC7945789

[3] Diallo BA, Usuf E, Ceesay O, D’Alessandro U, Roca A, Martinez-Alvarez M. Clinical research on COVID-19: perceptions and barriers to participation in the Gambia. BMJ Global Health. 2022;7(2):e007533. doi:10.1136/bmjgh-2021-007533. PMC8861886 35190459

[4] Mills E, Cooper C, Guyatt G, Gilchrist A, Rachlis B, Sulway C, Wilson K. Barriers to participating in an HIV vaccine trial: a systematic review. AIDS (London, England). 2004;18(17):2235–42. doi:10.1097/00002030-200411190-00003. 15577535

[5] Abraham TH, Deen TL, Hamilton M, True G, O’Neil MT, Blanchard J, Uddo M. Analyzing free-text survey responses: an accessible strategy for developing patient-centered programs and program evaluation. Eval Program Plann. 2020;78:101733. doi:10.1016/j.evalprogplan.2019.101733. 31675509

[6] Vanderslott S, Emary K, Te Water Naude R, English M, Thomas T, Patrick-Smith M, Henry J, Douglas N, Moore M, Stuart A, et al. Vaccine nationalism and internationalism: perspectives of COVID-19 vaccine trial participants in the United Kingdom. BMJ Global Health. 2021a;6(10):e006305. doi:10.1136/bmjgh-2021-006305. PMC8526520 34666989

[7] Vanderslott S, Palmer A, Thomas T, Greenhough B, Stuart A, Henry JA, English M, Naude RDW, Patrick-Smith M, Douglas N, et al. Co-producing human and animal experimental subjects: exploring the views of UK COVID-19 vaccine trial participants on animal testing. Sci Technol Human Values. 2021b;016224392110570. doi:10.1177/01622439211057084. PMC10387720 37529348

[8] Xin M, Luo S, She R, Chen X, Li L, Li L, Chen X, Lau J. The impact of social media exposure and interpersonal discussion on intention of COVID-19 vaccination among nurses. Vaccines. 2021;9(10):1204. doi:10.3390/vaccines9101204. 34696312 PMC8537317

[9] Vosoughi S, Roy D, Aral S. The spread of true and false news online. Science. 2018;359(6380):1146–51. doi:10.1126/science.aap9559. 29590045

[10] Kricorian K, Civen R, Equils O. COVID-19 vaccine hesitancy: misinformation and perceptions of vaccine safety. Human Vacc Immunother. 2021;18(1). doi:10.1080/21645515.2021.1950504. PMC8920251 34325612

[11] Bhopal S, Nielsen M. Vaccine hesitancy in low- and middle-income countries: potential implications for the COVID-19 response. Arch Dis Child. 2020;106:113–14. 32912868 10.1136/archdischild-2020-318988

[12] Betsch C, Ulshöfer C, Renkewitz F, Betsch T. The influence of narrative v. statistical information on perceiving vaccination risks. Med Decis Making. 2011;31:742–53. 21447730 10.1177/0272989X11400419

[13] GOV.UK. Celebrities get back to the ‘Rhythm of Life’ in new film supporting COVID-19 vaccination programme [online]. 2021. [accessed 2022 July 2]. https://www.gov.uk/government/news/celebrities-get-back-to-the-rhythm-of-life-in-new-film-supporting-covid-19-vaccination-programme.

[14] Vannette DL, Krosnick JA. The Palgrave handbook of survey research. 2017 Dec. p. 1–676. doi:10.1007/978-3-319-54395-6/COVER.

