# Supplementary material for: The collective voice of early phase COVID-19 vaccine trial participants: Insights for improving confidence in novel vaccines
Source: Hum Vaccin Immunother. 2023 May 3;19(1):2203023. doi: 10.1080/21645515.2023.2203023 (PMC10161940; doi:10.1080/21645515.2023.2203023)
Supplement: Supplemental Material [file KHVI_A_2203023_SM3889.pdf]

A

Statement		Strongly disagree % (n)	Disagree % (n)
**Altruistic motivations**			
**A**	I wanted to take action/make a difference/achieve something.	1.7 (6)	1.2 (4)
**B**	I wanted to help my community/society/country/world.	0.3 (1)	0.6 (2)
**C**	I wanted to advance the development of the vaccine.	0.30 (1)	0.3 (1)
**D**	I wanted to contribute to scientific progress	0 (0)	0.6 (2)
**E**	I wanted to contribute to the improvement of the health of others.	0 (0)	0 (0)
**F**	I felt a responsibility to take part	0.6 (2)	1.4 (5)
**Personal motivations**			
**G**	I wanted a reason to leave the house.	27.8 (97)	22.4 (78)
**H**	I felt that others would view my participation positively.	5.2 (18)	7.2 (25)
**I**	I thought that if I caught COVID-19 infection and needed to go to hospital I would have preferential treatment.	75.4 (263)	18.9 (66)
**J**	I wanted to tell others about my experience	25.8 (90)	23.8 (83)
**K**	I wanted to receive financial compensation	47.3 (165)	23.2 (81)
**L**	I wanted to report my experience to the [illegible]	83.4 [illegible]	14.3 [illegible]
